# Receptive Publics in Colonial Contexts: The Case of the Straits Philosophical Society

**DOI:** 10.1007/s11245-025-10203-6

**Published:** 2025-05-13

**Authors:** Lee Wilson, Natalie Alana Ashton

**Affiliations:** Department of Philosophy, VU Amsterdam, Amsterdam, The Netherlands

**Keywords:** Receptive publics, Counter hegemony, Colonialism, Public sphere, Resistance, Straits Philosophical Society

## Abstract

In cases where structural oppression conditions the broader public sphere, the democratic ideal of a receptive public may be threatened by at least two possible outcomes which appear to undermine its stated goal of increasing understanding of counterhegemonic ideas amongst mainstream, oppressive groups. Either (a) counterhegemonic ideas are defanged to make them sufficiently palatable to a new audience, or (b) counterhegemonic ideas are taken up intact, and as a result the extant networks of publics which depend on oppressive structures and hierarchies will be destroyed. As we will argue, in certain cases of colonialism such as the Straits Philosophical Society in colonial Singapore, the conditions which receptive publics are supposed to ameliorate: (i) the social costs of speech, (ii) inequality of epistemic labour, and (iii) the antagonism between groups, are not only an irreducible feature of counterhegemonic efforts, but are in fact increased in the attempt to maintain receptive publics. However, this may be more a feature than a bug: receptive publics need not be seen only as communicative intermediaries for oppressed groups, but as a possible dialectical step towards new modes of socio-material life.

## Introduction

In recent years, much scholarship—philosophical and otherwise—has focused on problems of political fragmentation, the idea that discourse has become characterised by identity politics, and a pressing need to communicate across different experiences of oppression (e.g. Squires 2002; Burrows 2014; Mothsaathebe 2018; cf. Deumert 2019; Aikin and Talisse 2020; Dutilh Novaes 2020; Tanesini 2020; Broncano et al. 2021; Dommett and Verovšek 2021; de Ridder 2021; Jackson and Kreiss 2023; Hannon 2023). Habgood-Coote et al. (2024) have sought to shift the focus of this discussion from *speech*, and what is *said*, to *listening*, or how that speech is *received* and understood, with the concept of receptive publics.

Whilst we welcome the shift to looking at the responsibilities of the listening party—and specifically when that party is the one with greater social power—we aim to show that, in at least some contexts, the obstacles to productive listening are greater, and of a different kind, than Habgood Coote et al. account for. To make their argument, Habgood-Coote et al. draw on two main areas of literature: the literature on the public sphere, and that on contemporary social epistemology. In this paper, we will bring this work into conversation with a further area: the historical archive and philosophical literature on colonialism.

Exploring historical instances of receptive publics, specifically those in *colonial contexts*, show that being bad at listening is not just a coincidental by-product of oppression, it is often a function of it. In this paper, we will focus on the dynamics of one such instance with the Straits Philosophical Society (1893–c.1921) as our case study, a coloniser-led learned society in *fin-de-siècle* Singapore (the administrative capital of the British Straits Settlements) whose object was “the critical discussion of questions in Philosophy, History, Theology, Literature, Science, and Art” (Rules of the Straits Philosophical Society 1910), setting out to draw on “the relative powers of the minds of the various nationalities in Singapore” after “so many generations in particular countries and callings,” including the “different powers of observation developed” by the “Tamils, Malays, and Chinese” (Warren 1893, 9).

In the next section we will begin by briefly introducing the concept of the public sphere, and two key moves in the surrounding literature: the first is to recognise that there is a plurality of different public spheres, and the second is that these various spheres are most usefully distinguished by their function, rather than merely by the identity of those who occupy them.

In section 3 we will outline the target of this paper: receptive publics. These are a subtype of public sphere spaces, ones where people who don’t experience a particular form of oppression can go to learn about that oppression from people who do experience it. In line with the literature surveyed in section 2, we focus on the two proposed functions of these spaces (developmental and amplificatory) and, correspondingly, two ways in which such a space can be said to have failed.

In section 4 we consider the expropriative nature of colonial contexts and highlight the significance of this to the idea of receptive publics, attending to the case of the Straits Philosophical Society as one accordingly failed attempt to realise it within the context of colonial Singapore. In section 5, with this in mind, we diagnose the frustration of receptive publics in colonial contexts by discussing two challenges for them; presenting a dilemma for each of receptive publics intended functions. The *developmental challenge* turns on the fact that the counterhegemonic ideas receptive publics are supposed to create space for are aimed at eliminating the relative power and privilege of the group receiving the information. In colonial contexts, this means the power of a group who are not colonised, and instead are likely to be the colonisers. In such a situation, there’s a high risk that the audience will either commit a type 2 failure—by interpreting the counterhegemonic ideas in such a way that their position seems to them not to be at risk—or will correctly understand both the ideas, and their incompatibility with their own position as coloniser, and will react badly to the counterhegemonic ideas. If this dilemma is avoided and the developmental function is somehow successfully fulfilled, these same problems arise at a second level in the *amplificatory challenge*: when the powerful audience attempts to communicate the counterhegemonic ideas they have learned to other groups—perhaps to even more powerful figures, more central to the colonial situation—the ideas may again may be misinterpreted, or trigger a backlash.

In the concluding section, we briefly explore the consequences of this argument and what it tells us about the potential of receptive publics in colonial contexts. Ultimately, we suggest that even if they fail to facilitate productive listening, receptive publics may have an intermediary, dialectical role, on the way to more radical socio-material changes.

## The Public Sphere(s)

The concept of *receptive publics* (Habgood-Coote et al. 2024) is a recent addition to the extensive ‘public sphere’ tradition, made famous by Jürgen Habermas (1989). As the problems we identify with receptive publics have their roots in this early work, and our proposed solutions build on existing critiques, we will begin by highlighting some key moves in this literature.

The ‘public sphere’ is the name given to forums for political discourse outside of formal, representative institutions. It encompasses spaces where ‘the masses’ can meet on a supposedly equal footing, in order to discuss issues of common concern, subject the decisions and actions of the authorities to ‘public reason’, and in doing so—supposedly—hold them accountable. The public sphere is sometimes referred to more colloquially as meeting in the ‘town square’ or the ‘public square’, in reference to the classical example of the ancient Greek agora—a marketplace where citizens would meet daily, to discuss social and political issues alongside buying and selling. Habermas himself focused on the function of seventeenth century coffee houses and salons in Europe, and more recently some have referred to social media sites—in particular the microblogging site Twitter^1^—as ‘the digital public square’ to indicate their centrality as a public location of political and social discourse.

This concept has been incredibly influential. Nancy Fraser says that “no attempt to understand the limits of actually existing late capitalist democracy can succeed without in some way or another making use of it” (Fraser 1990, p. 57). However, it has also been heavily criticised, most famously by Fraser herself (Fraser 1990. See also, e.g., Eley 1987; Landes 1988; Ryan 1990). She is sensitive to the way that Habermas uses the concept of the public sphere as both a description of actual and historical spaces, and as an ideal that we might strive to create, and how doing this obscures serious limitations with his account as formulated. Namely: Habermas’s original account gravely underplays the role of oppression in shaping discourse and discourse spaces. When wielding this—arguably indispensable—tool it is therefore important to be very clear about how to deploy it critically. Only then can we avoid repeating Habermases’ mistakes, and adequately illuminate current areas of political discourse.

Fraser points out that Habermas failed to properly acknowledge how marginalised groups (she focuses on women and the working class) were and are excluded from actually existing public spheres (Fraser 1990, pp. 59–60). This exclusion limits the range of topics that are considered legitimate for discussion, and so undermines the supposed purpose of holding powerful authorities to account. Similarly, Fraser argued that Habermas failed to recognise the existence of alternative discursive spaces (which she calls subaltern counterpublics) where these marginalised groups could, did, and do meet to discuss issues of common concern to them (Fraser 1990, pp. 60–61). Her examples of subaltern counterpublics include bourgeois, nineteenth century, women’s-only philanthropic organisations, which mirrored and sought to influence the main (bourgeois, male) public (Fraser 1990, p. 61) as well as a late-twentieth century feminist movement in North America, encompassing a “variegated array of journals, bookstores, publishing companies, film and video distribution networks, lecture series, research centers, academic programs, conferences, conventions, festivals, and local meeting places” (Fraser 1990, p. 67).

Since then, Catherine Squires (2002), has synthesised the work of other feminist and African-American scholars to make further, important additions to this picture. She points to a wider range of subaltern counterpublics with more diverse functions, emphasising that counterpublics can influence the public sphere through strategies such as boycotts and civil disobedience. She also sketches out two other types of counterpublic space: *enclaves* are groups that produce counterhegemonic ideas and liberation strategies, but have to hide them from the main public for survival or to avoid sanctions—for example groups of African Americans organising under and against Jim Crow laws (Squires 2002, pp. 448 and 457–459). And *satellites* are groups which prefer to be separate, and so distance themselves from the public sphere, in order to maintain a distinct identity, but which may be involved in wider public discourses from time to time. Her example here is of the Nation of Islam (Squires 2002, pp. 448 and 463–464).

This leaves us with two important considerations to bear in mind when working with the concept of ‘the public sphere’. First, following Fraser, we should adopt a pluralistic picture of ‘the’ public sphere. Rather than occupying a single domain for social and political discussion, citizens generally find themselves spread between various spheres of debate (also see Asen 2018 on this point). These may differ in their demographic composition [think, for example, of Black Twitter as a distinct counterpublic from ‘the’ main digital public square (Graham and Smith 2016)] but also, importantly—and as Squires pointed out—their function. These two points are crucial to understanding both what the role of receptive publics could be, and how—as we will argue in section E—that role might be self-defeating, at least in colonial contexts.

## Receptive Publics

With this groundwork laid, we can now introduce the subject of this paper: receptive publics. Habgood-Coote et al. have analysed the contemporary understanding of the phenomenon of ‘cancel culture’ in terms of multiple publics (Habgood-Coote et al. 2024). They posit that the rise of social media (alongside other social and political changes) has led some groups which previously functioned as enclaves to function instead as counterpublics, and so to go from hiding their counterhegemonic ideas to attempting to use them to influence ‘the’ main (or, more accurately, the range of more dominant) public(s). Prominent examples include the #MeToo movement, and Black Lives Matter.

Habgood-Coote et al. posit that this shift has contributed to an increase in inter-group friction. This is sometimes—typically from the perspective of dominant groups—described as ‘cancel culture’—(the idea that there are very high social costs for speech which flouts a narrow and recent set of social norms) (Habgood-Coote et al. 2024, p. 114). Whilst a range of corresponding effects on marginalised groups have been highlighted by social epistemologists, such as: epistemic injustice (Fricker 2007); epistemic oppression (Dotson 2014); and epistemic exploitation (Berenstain 2016). Habgood-Coote et al. attribute this state of affairs to a more general problem, one they call ‘the Listening Problem’: a failure to appropriately receive and understand concepts of marginalised and minority groups, due to the fact that public discourse and its supporting institutions are “organised around the perspectives and interests of dominant and majority groups.”

Receptive publics are proposed as a solution to this problem. They can be thought of as a composite public, containing members of the saliently oppressed group, as well as sympathetic members of groups which are not saliently-oppressed. The idea is that these spaces—which the authors recognise exist already, in varying, often imperfect, forms—create opportunities for members of oppressed groups to explain their experiences of oppression, and for members of groups which are not saliently-oppressed to learn about forms of oppression which they don’t experience.

These spaces must, Habgood-Coote et al. argue, be designed to minimise or ameliorate three barriers to listening which they say are characteristic of intra-group discussion elsewhere (loosely mapping onto the values of *egalité*, *liberté*, and *fraternité*):

- unequal distribution of epistemic labour, i.e. the work of moderating tone, language and etiquette, as well as managing the epistemic friction and psychological conflict that one’s interlocutors experience;
- the perception (and/or actuality) of high social costs (or ‘cancellation’) associated with speaking inexpertly on topics that one is not well-versed in;
- antagonistic communication

In an ideal receptive public these problems would not be prohibitive, as the not-saliently-oppressed would be aware of the likelihood of feeling threatened, and would “put that friction to work” to develop more nuanced understandings (Habgood-Coote et al. 2024, p. 138); they would recognise inequalities in epistemic labour and strive to be responsible, active listeners (Habgood-Coote et al. 2024, p. 138; Notess 2019); and they would endeavour to be the kind of audience which “is willing to put in interpretative work” and “pursue strategies of communication that do not assume a basic level of hostility” (Habgood-Coote et al. 2024, p. 139). They hope that conceptualising these publics will support the creation of new ones, or the better maintenance and management of those that already exist.

Following Squires, we think it is most helpful to define different publics in terms of their functions. As we saw in the previous section, the function of ‘the’ main public is to discuss matters of common concern and to hold power to account, whilst the function of counterpublics is to generate counter discourses that fill in the gaps in the main public(s), and to influence them to take these counter discourses on. Accordingly, we can understand the broad role of receptive publics as supporting the understanding of these counter discourses in more dominant publics, but more specifically this can be broken down into two distinct functions, which we will call the *developmental* and *amplificatory* functions.

The *developmental function* of receptive publics is to develop the dominant members’ understanding of the kind of oppression that the receptive public is focused on, as well as the skills that are needed for receptivity/listening (Habgood-Coote et al. 2024, p. 130), whilst the *amplificatory function* is to support the influencing of dominant publics, by sharing, repeating, and backing up counterhegemonic ideas with other members of these publics (cf. Habgood-Coote et al. 2024, p. 133).

In the next section we will discuss some challenges that colonial contexts present to these functions. But before moving on, we should take a moment to explain one more concept related to receptive publics, namely: *false* receptive publics.

Habgood-Coote et al. characterise a false receptive public as a group that presents itself as a receptive public, but which cannot achieve the developmental or amplification goals of receptive publics, either because it is constructed around, and seeks to create receptivity for, a group which is “not composed of members of an oppressed group” or because it “non-accidentally [fails] to produce counterhegemonic discourses”. In what follows, we will refer to the failure of (apparent, but false) receptive publics to achieve developmental and amplificatory goals for these reasons as ‘Type 1 Failures’. But we will also highlight a second way in which a receptive public can fail at its developmental and amplificatory goals. A ‘Type 2 Failure’ is when a receptive public is constructed around a genuine counterpublic, but the members who are not saliently-oppressed systematically fail to learn about counter hegemonic ideas or develop listening skills.

## The Expropriative Nature of Colonial Public Spheres

The thrust of Fraser’s critique, above, was that public discourse in “modern stratified societies characterised by exclusionary public spheres” should aim not at a single, unified public, “but at a network of connected publics” (Habgood-Coote et al. 2024, p. 127), whilst Squires emphasised that these publics fulfil a diverse range of functions. In conceptualising receptive publics, Habgood-Coote et al. identify an additional type of public which they characterise by the developmental and amplifactory functions explained above, and by the goals of seeking to ‘influence the public sphere’ and ‘ameliorate marginalisation in the public sphere.’ We will suggest that—at least in some public spheres—these goals are too conciliatory. This is most clear in the case of colonial publics, i.e. publics that are embedded colonial systems (e.g. the British Empire).

The problem in at least some such public spheres is not that oppressed groups are excluded per se, but that the very formation and maintenance of such spaces systematically perpetuates, and they themselves are constructed by, the oppression of certain groups.

For example, Habermas’ coffee houses, seen as crucial for capitalism’s triumph over feudalism, required the non-epistemic labour of those “[w]omen of a less exalted social status [who] found it much easier to enter a coffeehouse than their genteel sisters, not least because the majority of them were there to serve the male patrons” (Cowan 2005, p. 250). And, as media theorist Wendy Willems has recently noted, at the centre of the British Empire, “coffee houses and newspapers [in London] were intricately linked to the slave trade and slavery in a number of ways”:

First of all, in the most direct way, they facilitated the sale and recapturing of enslaved people. […] Coffee houses were often listed as point of contact for prospective sales. […]

A second more indirect connection pertains to the activities that the customers of coffee houses and readers of newspaper were involved with. In many ways, they represented the new commercial elites who benefited from the slave trade. […] the coffee houses also hosted networking opportunities for commercial elites involved in insuring slave ships. […]

A third link relates to the way in which the coffee houses acted as spaces where colonial commodities like sugar, which were produced in slave-based economies in the Caribbean, were consumed. […] those consuming sugar also were able to do so as a result of the violence that enslaved people were subjected to on the plantations. (Willems 2023, 20–21)

That is, such institutions, *qua* sites defined by such commodity consumption, were existentially dependent on products of colonial expropriation (e.g. coffee beans, sugar) that involves, but is not limited to, land-grabbing, the slave (and later coolie) trade, as well as ecological destruction (see Arifin 2023; Combrink 2021, Marquese 2022). The particular social structure of the coffee house—which Habermas would regard as foundational to the public sphere writ large—was therefore not only *causally constructed* by colonial expropriation, being a structure that came into being due to expropriation, but also *constitutively constructed* by colonial expropriation, being a structure that functioned to uphold the status of white, bourgeois, imperial elites and shaped the emergent ideas that echoed accordingly therein—such as John Locke’s notorious positions on land-grabbing and slavery in the British colonisation of America (Farr 2008; cf. Haslanger 2003).

In such situations, the exclusionary nature of such colonial publics is neither due to “incidental departures from the constitutional ideals of [public] spheres” nor a result of misrecognising counterpublics (Habgood-Coote et al. 2024, p. 125), as much as they are features of the colonial public itself. As Fraser herself more recently observes, “the subjection of those whom capital expropriates is a hidden condition of possibility for the freedom of those whom it exploits [with the mediation of a wage contract]” (Fraser 2016, p. 166). Expectedly then, the causal and constitutive construction of (at least some) colonial publics by expropriation may also be found in peripheral spaces within the broader imperial public sphere established by a pan-imperial network of publishers and readers (see Raymond and Moxham 2016).^2^ Our focus here is on one such case, approaching the Straits Philosophical Society additionally as a *receptive* public—albeit ultimately a false one. Since it is unlikely that our readers would be familiar with this particular piece of intellectual history at the colonial peripheries, we will now outline in some detail the historical aspiration and failure of the society as, effectively, a receptive public for Sinophone counterhegemonic ideas in the public sphere of the British Empire.

Singapore, which became a British colony in 1819, presented a particular politico-epistemic conundrum for its colonial administrators, who founded it with an “‘enlightened’ approach to empire,” drawn from “Enlightenment ideas of progress and liberalism” on “criticism of the mercantilism of the Dutch” (Lim & Brophy 2023, p. 12). The administration’s commitment to the colony’s status as a free port, alongside the plantations they set up in both Singapore and the Malayan states (to compete with the Dutch in Southeast Asia), led to an influx of Chinese migrant labourers to the British Straits Settlements—such that they became the majority ethnic group in Singapore by the 1840s. These Straits Chinese migrants were overwhelmingly wage- or day-labourers (including many ‘coolie’ labourers and sex workers) from Southern China, who did not receive any formal education—much less an anglophone one. Furthermore, due the Flint Affair in 1759 in China,^3^ as a consequence of which the Qianlong Emperor banned the teaching of Chinese languages to foreigners on pain of death (until the Treaty of Wanghia in 1844), the initially India-based colonial administration and anglophone merchants neither had the experience nor the linguistic and cultural knowledge to govern and organise this burgeoning population. Colonial administrators and merchants in Singapore thus found themselves having to rely on a small but significant portion of (Peranakan) Chinese migrants who came instead from the surrounding archipelago—e.g. Malacca, Riau—with pre-existing capital and an eagerness to learn the English language for trade.^4^ This group (the *babas*) effectively became—and also saw themselves as—intermediaries for the wider Chinese populace and British colonisers (Teo 2019).

Newspaper presses, e.g. *The Singapore Free Press* and *The Straits Times*, and publishing houses flourished as a result of a broad commitment by administrators to liberty of the press (after a gagging act in the colonies was lifted in 1835). And with the inward circulation of newspapers like British-based *Financial Times* and *The Guardian*, in addition to the outward circulation of local publications within the empire, Singapore quickly became integrated into the imperial public sphere. That said, the local presses were hardly representative of the concerns of the colony’s largest population for about a century, until the establishment of the *Malaya Tribune* in 1913, which aimed “[t]o represent the views and interests of the Asian communities” (Wong 1951, p. 2).

We can now understand more of the colonial motivations surrounding the founding of the Straits Philosophical Society in 1893 (see also Lim & Brophy 2023; cf. Jose 1998), as well as the introductory address of the first president of the Straits Philosophical Society,^5^ quoted at the outset of this paper, that it “would be a very worthy and proper object for a Philosophical Society to have in view” the development of “the relative powers of the minds of the various nationalities in Singapore” (Warren 1893, p. 10). He specifically highlights an interest in knowing whether the supposition that “the Chinaman [*sic*] is a very accurate and close observer […] extends to all matters in everyday life” and also a desire “to ascertain how [the Chinese person] first exercises these faculties, i.e. whether they are cultivated by his parents or whether his own innate thriftiness induces their cultivation,”—noting further that such an undertaking by the Straits Philosophical Society “would be the case of Singapore setting the example of doing what will certainly have to be undertaken at home in a few years” (*ibid.*, 9–10).

Apart from the core members of the Straits Philosophical Society, the society otherwise had a pan-imperial membership and readership: originators of essays and criticisms in the extant record of the society’s proceedings include residents in not only Singapore but also London, Edinburgh, Ceylon, South Africa, and the rest of British Malaya. This was organised by an elected Executive Committee, consisting of “a President, a Secretary, and three other Members who [were] elected annually” (Rules of the Straits Philosophical Society 1910, p. 1). And while in its early years “discussions following the talks were designed to institutionalize ‘freedom of thought and expression,’ outside of the gaze of the colony’s developing public sphere,” contributions were reproduced in print elsewhere: e.g., with an anthology of earlier contributions compiled by the society’s then-president in 1913 or with “proceedings of subsequent years 1911–1916 […] made available in pamphlet form by the Methodist Publishing House of Singapore” (Lim and Brophy 2023, pp. 5–6). Contributions between the Straits Philosophical Society and the Straits branch of the Royal Asiatic Society also sometimes overlapped.

Given the aforementioned context and a broadly liberal orientation of the colonial administration (Jose 2010), that the first president of the Straits Philosophical Society would aspire for it to effectively become a *bona fide* space that was particularly receptive to the epistemic contributions of the Chinese population—i.e. a receptive public—is thus unsurprising. In fact, of the members (exclusively men) constituting the society throughout its years, only two core members were Chinese: Tan Teck Soon (1859–1922) and Lim Boon Keng (1869–1957). Both men, each internationally reputed then for their knowledge of Chinese philosophy and culture, were simultaneously involved in a broader Straits Chinese counterpublic through various community initiatives, such as the Amateur Drawing Association or the creation of or contributions to the *Straits Chinese Magazine*. The latter was a quarterly anglophone periodical that not only centred the voices of the Straits Chinese but also included contributions by their colonisers in an effort to promote mutual understanding (Holden 1998; Fermanis 2021). Contributions by the Straits Philosophical Society were also sometimes reprinted in the magazine.

However, it should be underscored that the construction of the Straits Philosophical Society as a public space in 1893 was also due to the wealth accumulated by the colonial administration in the preceding decades—most notably through the opium trade which was undergirded by the ‘coolie’ trade. As historian Carl Trocki notes, “[i]t is difficult to see how the British could have taken control of the commerce of India, China, and Southeast Asia in less than half a century without opium” as “the major form of economic leverage” (Trocki 1990, p. 221). Such expropriation was, furthermore, often a self-conscious enterprise: “Anglophone capitalists understood the close connection of contraband opium to Chinese captive labor, cynically linking them as ‘poison and pigs’ […]—their cash cows” (Driscoll 2021, p. 5). And as set out in its rules, the society would meet monthly for dinner in a hotel—and the only extant photograph of the society locates them at the table of the most prestigious Singapore Club, a site that gathered the chief economic and political players in the colony (Makepeace et al. 1921, p. 302). Moreover, membership was restricted to graduates from recognised (Euroamerican) universities, “[f]ellows of a recognised Learned or Scientific Society,” or otherwise deemed by the society’s members to be “of distinguished merit […] in any branch of knowledge” (*Rules* 1910, 2). Coupled with a $5 entrance fee, $25 annual fees (increased from $15 in 1893) and fines for absentees, at a time when average real wage was $93–111 among a predominantly impoverished population (Choy and Sugimoto 2018), the makeup of participants was restricted to a particular socio-economic class. Society discussions often reflected this: for example, the withholding of applying liberal principles to ‘oriental dependencies’ or negative eugenics.

We can thus anticipate how, while Tan and Lim were able to (at least sometimes) express counterhegemonic ideas to their colonial interlocutors, the record of the criticisms or discussions by these interlocutors following their contributions evidence a continued lack of understanding. The goals of an ideal receptive public of solving the listening problem—in addressing (i) unequal distribution of epistemic labour, (ii) the perception of high social costs speaking inexpertly on unfamiliar topics, and (iii) antagonistic communication—were frustrated by their colonial context (and *systematically so*, as we shall argue in the following section).

(i) Both Tan and Lim often had to work to temper their tones when criticising the colonial administration—with an eloquence and mannerism befitting of an ‘English gentleman.’ (On rare occasions, this temperance slipped to deleterious effects on the discussion.) Yet, Tan’s consistent attempts to bring the grievances and contributions of the disadvantaged Straits Chinese population in Singapore (e.g., women sex workers and labourers, whom he passionately defends elsewhere) to the attention of the colonial administrators in audience still often went unappreciated. Tan’s essay analysing Chinese local trade (now cited by historians as an authoritative contemporary source) was even denounced by his critic as unworthy of the society’s time altogether (Allinson 1901).
(ii) Criticisms were also launched by Tan of their colonial interlocutors’ “feeble and spasmodic attempts […]to understand and comprehend the natives who surround [them] on every side” (Tan 1902, p. 63): such as the colonial members’ avoidance of presenting any essay on Chinese philosophy, religion, and culture (except *one* time, in the absence of both Tan and Lim). Criticisms and discussions on these topics (almost always prefaced with protracted disclaimers about their ignorance and inexperience) were otherwise only prompted by the two Chinese members—and were often only tangentially related. Lim was recorded responding to the discussion following his essay on “The Influence of Religion in China,” wherein his interlocutors meandered about concerns of the religious beliefs of the *babas*, the incapacities of the Chinese student of morality, and the Christian deity:
  He [Lim] had advanced a number of views in hopes of obtaining additional information regarding the influence of foreign religions upon each other in early times and regarding the development of religion in China, but the discussion had centred round other points, and no information had been given to elucidate the question in which he was most interested. (Wilkinson 1895, 74)
(iii) Moreover, Lim’s attempt to introduce the idea of a “tendency to over-legislation” in an essay was summarily rebuffed by his critic as not only non-existent in the colony but nonsensical—with the latter asserting that “we want more legislation, and less consideration for individual rights” (Ritchie 1906, p. 80). Meanwhile, a similar critique made by Tan concerning British discriminatory restrictions on higher offices in the Chinese-controlled Imperial Maritime Custom Service was met with outrage and disbelief at the lack of appreciation for the Empire’s efforts to improve the world (Thomas 1907, see also Wilson 2025). The critic of Tan’s aforementioned essay on Chinese local trade would even conclude with a dehumanising (yet common) comparison between Chinese people and dogs. The reception of Tan and Lim’s counterhegemonic ideas within the space of the Straits Philosophical Society was thus continually frustrated by the defensiveness of their interlocutors with respect to the colonial situation (cf. Jose 1998).

With this case in mind, we will now go on to argue that the failure of the receptive publics in colonial contexts, such as the Straits Philosophical Society, is due to the fact that, at a more fundamental level, aiming to ameliorate ‘barriers’ to understanding, such as the inequality of epistemic labour, social costs of speech, and antagonism between groups, may be an inappropriate goal for networks of publics and associated counterpublics so conditioned by such structural oppression. Such goals would either maintain the dominant centre of the network, around which counterpublics are organised, or overlook the centre’s structural competitive advantage, given its expropriation of counterpublics’ labour, property, and/or persons. We will explain this dilemma in more detail in the next section.

## Two Kinds of Challenges for Receptive Publics

If we do think that such expropriative dependencies are constitutive of at least some public spheres and networks—such as those indelibly linked to colonialism—then there are two kinds of challenges corresponding to the two constitutive functions of receptive publics that are meant to mediate these networks: developmental challenges and amplificatory challenges. These two kinds of challenges ultimately either threaten a receptive public with a collapse into a false receptive public, inasmuch as the colonist would “systematically [fail] to learn about counterhegemonic ideas or to develop listening skills,” and thereby (a) defang counterhegemonic ideas; or, as a sort of anti-colonialist might hope, (b) threaten to destroy the extant network of the public sphere and connected counterpublics.

### Developmental Challenges

The education of a privileged portion within a receptive public (hereafter *Pr*) is meant to mitigate some of the social costs for both *Pr* and the marginalised portion (hereafter *Mr*). However, a knowledgeable privileged group in the broader public sphere (hereafter *Pp*) is not ultimately the *end* of counterpublics in general (hereafter *Mc*)—especially in anti-colonial contexts. A knowledgeable, and even benevolent, colonial government remains a colonial government. Developmental challenges thus largely arise from how counterhegemonic ideas in the receptive public (at least partly) aim at eliminating the very position of *Pr*. This existential threat to privileged listeners results in a political quandary identified by Albert Memmi as ‘*the Coloniser who Refuses*.’

In the ideal case where epistemic friction is managed in the conveyance of counterhegemonic ideas, a dilemma is occasioned for *Pr* in colonial situations:

*either* (Da) *Pr* “[likens] the colonial situation to any other and therefore [applies] to it the same analytical methods, judging it and the colonized in accordance with [*Pp*] traditional values” that corresponds to *Pr*’s political situation as a member of the colonising culture;

*or* (Db) *Pr* “must consider the colonial juncture as being original and abandon [*Pp*] values and usual habits of political thought which induced [them] to take sides” (Memmi 1957, 77).

With (Da), *Pr* would risk either defanging counterhegemonic ideas as they assimilate such ideas into their extant, hegemonic conceptual scheme [i.e. a Type 2 Failure]. This is a bigger problem than Habgood-Coote et al. noted when they attended to the appropriation (or expropriation) of counterhegemonic ideas. Often, counterhegemonic ideas target *fundamental* hegemonic ideals—thought to be precisely the animating ideals of expropriation—that, should these fundamental ideals not be unseated, necessitates incoming ideas to be appropriated accordingly. Tommy Curry has noted an “epistemic convergence [of African American philosophy] with white philosophical traditions,” with “historic Black thinkers [made] *safe for white consumption* by reading the importance of race and the centrality of culture out of Black thought”—e.g. “W.E.B. Dubois is read as the Black Hegel, the Black James, the Black Dewey, and Frantz Fanon as a Black Sartre or Husserl” (Curry 2011, 315–6). As Curry observes, following Sylvia Wynter, this is due to how, “insofar as Blacks are human or claim their humanity,” they must always appeal to an “always already” fixed ideal of “European *humanity* believed to be universal” which “Black’s humanity” are presumed as “analogous with […] regardless of its anthropological assumptions” (Curry 2011, p. 323, *emphasis added*). Similarly, attempts to comprehend Confucianism and Daoism by colonial interlocutors in the Straits Philosophical Society often relied on reductive comparisons that mapped Chinese philosophers too quickly onto those with which they were more familiar, such as “Confucius [being] the Aristotle, [Laozi] the Plato of China” (Lamont 1895, p. 66; cf. Kirloskar-Steinbach and Kalmanson 2021).

Notably, Tan and Lim themselves also often engaged in a similar comparatism when expounding on Chinese-philosophical concepts to an anglophone audience—coloniser or colonised. Tan, for example, in introducing Chinese religions to his interlocutors, described ‘Dao’ “in Kantian phraseology” as “the category of the ‘a priori’ or the ‘purely formal’,” albeit still being careful to distinguish between the “more abstract and intellectual” nature of the “European philosopher’s representation” and the “more concrete and objective” one of “the Chinese” (Tan 1912, p. 98). Characterising Daoism as “a system of transcendental philosophy,” Tan further admitted that its “ontological speculations are considered by some to be Hegelian in their main tendency” (*ibid.*).^6^ As literary scholar Porscha Fermanis notes in her examination of Tan and Lim’s contributions to the *Straits Chinese Magazine*, “Lim and Tan [saw] comparatism as a means of empowering Chinese civilisation within an increasingly racialised imperial world-system” (2021, p. 361). This, however, remains in tension with how “the magazine is conventionally read as an apology for both British and Chinese imperialism,” where “loyalism and participation in the colonial public sphere is seen as false consciousness, at odds with the more authentic forms of radicalism associated with workers and peasants in the kampongs.” (p. 371).

Whether this predicament was indeed true or still true of the field of Black studies or anglophone Chinese philosophy more generally is not for us to assess here, but the threat of such epistemic convergence is nevertheless clear for receptive publics under conditions where a fundamental hegemonic ideal is held fixed as a polar star in extant epistemic institutions. What is especially crucial for us here is that, as Memmi observed, unseating a fundamental hegemonic ideal like ‘humanity, as conceived by Europeans,’ may mean undercutting *the very motivation behind* Pr*’s political solidarity*—that is, why *Pr* would participate, or continue participating, in a receptive public—to begin with.

With (Db), *Pr* would find that the social costs of listening are much higher than merely “backlash for misstepping” (p. 118)—the existential threat to their fundamental ideals is not incidental to the learning process of receptive publics but the very *ends* of these publics. Although an obstacle that may be nonetheless overcome, the psychological demand of such a process on *Pr* may perhaps be most poignantly put in T.S. Eliot’s own reflection on his encounter in his study of Indian philosophy at the start of the twentieth century:

Two years spent in the study of Sanskrit under Charles Lanman, and a year in the mazes of Patanjali’s metaphysics under the guidance of James Woods [1911–1914 at Harvard], left me in a state of enlightened mystification. A good half of the effort of understanding what the Indian philosophers were after—and their subtleties make most of the great European philosophers look like schoolboys—lay in trying to erase from my mind all the categories and kinds of distinction common to European philosophy from the time of the Greeks. My previous and concomitant study of European philosophy was hardly better than an obstacle [1910–1916 at Harvard, Sorbonne, Oxford]. And I came to the conclusion—seeing also that the ‘influence’ of Brahmin and Buddhist thought upon Europe, as in Schopenauer, Hartmann, and Deussen, had largely been through romantic misunderstanding—that *my only hope of really penetrating to the heart of that mystery would lie in forgetting how to think and feel as an American or a European: which, for practical as well as sentimental reasons, I did not wish to do*. (Eliot 1934, 40–41, *emphasis added*)

While it is unclear whether a similar psychological threat presented itself to the unreceptive colonisers of the Straits Philosophical Society, the fear of ‘forgetting how to think and feel as *X*’ was not foreign as a concept to them. Lim made an inverse point about the cost of Christianisation for Chinese people in his debut essay, that “the missionary has not sufficiently realised […] a Chinaman can scarcely be a Christian in China without becoming almost completely denationalised,” going further to claim also that “[t]he very social fabric of China must be broken up if the Chinese as a whole become Christian” (Lim 1895, p. 62). Lim himself would have been intimately familiar with Eliot’s ‘practical as well as sentimental reasons,’ having been baptised in a church during his medical studies in Edinburgh just a couple of years earlier—though publicly moving away from Christianity in favour of Confucianism altogether after this essay.

Yet, it is clear that *some* degree of commensurability between the ideals of the sympathetic members of a receptive public who are not saliently oppressed and the ideals of the saliently oppressed members is a condition of the very possibility of a receptive public. This was a position otherwise affirmed by Tan and Lim in their efforts to communicate their ideas. In the former’s 1898 review of Paul Carus’ *Buddhism and Its Christian Critics* for the *Straits Chinese Magazine*, for example, Tan remarked on an overall progression in the broader European reception of Chinese ideas: from the translations of “the French savants Abel Remusat [Jean-Pierre Abel-Rémusat] and Stanislas Julien” which formed “a new and more favourable opinion […] as regards the utility of Chinese literature and the capabilities of the Chinese race”; to “the basic elements of Chinese civilization [being] revealed and to some extent appreciated” by “scholars such as Legge, [Samuel] Beal, [Thomas] Watters, and [Herbert A.] Giles”; and to “the philosophic studies of scholars of the New World [such as Carus]” providing “the fairest estimate of Chinese characteristics but also […] the deepest insight into Chinese faith and beliefs”—the last of which Tan hoped would “transcend all racial distinctions and eventually re-enlighten even ourselves [i.e., the Straits Chinese]” (Tan 1898, p. 31; cf. Fermanis 2021).

However, what was clear that would have undergirded (Db) among some members within the Straits Philosophical Society who were not saliently oppressed was a dominant assumption of a *categorical* incommensurability that echoed Kipling’s sentiment that ‘East is East, and West is West, and never the twain shall meet.’ We can find different kinds of such incommensurability articulated: e.g., *foundational incommensurability*, where “the foundations that traditions use to make sense of the world around them are so different from one another that members of these traditions cannot understand one another”; *linguistic incommensurability*, where “philosophical traditions from different cultures depend on distinctive languages that cannot be translated into one another” (Connolly 2023, p. 72).^7^ The 1907 presidential address to the society, by a then-newly elected Henry N. Ridley, put forward a position of foundational incommensurability between ‘East’ and ‘West,’ coupled with a more-than-tacit declaration of his preference for his ‘own’ tradition and ideals. Ridley noted that while “East and West are meeting on physical grounds” and “[the Orientals] may graft the best parts of the Western civilization on the best of the Eastern, and so further the development of the human race” (with the address neglecting the possibility of the converse), “Oriental and Occidental will remain distinct to the end of time” (1907, pp. 11–13).

And, just a decade before, Straits Philosophical Society member Archibald Lamont (1864–1933) gave voice to a concern for linguistic incommensurability in a direct contribution of his to the *Straits Chinese Magazine*. (Whether Lamont actually believed the position he put forward is unclear from the contribution.) Lamont was a graduate in ethics from the University of Glasgow, a Presbyterian minister, and had been Tan’s collaborator on the 1894 novel *Bright Celestials: A Chinaman at Home and Abroad*, as well as *The Daily Advertiser*, which they co-owned and ran together in the same year. Lamont’s contribution to the *Straits Chinese Magazine* was a fictional dialogue between European & Straits Chinese spiritualists and the ghosts of Shakespeare & Confucius in a séance, in which “Shakespeare stands as a symbol for ethnocentrism on a grand scale, as well as for the ways in which western literary classics lose their meaning and value outside of a European context” and “Confucius fares little better” (Fermanis 2021, p. 369). Fermanis notes that “the story ends without any sense of agreement between the Straits Chinese and their British friends, pointing to the incommensurability of their religious beliefs and their positions as coloniser and colonised, as well as to the failure of either Shakespeare or Confucius, as representative figures of a western and eastern canon, to adequately speak to or reconcile those positions” (*ibid.*).

Both these horns, (Da) and (Db), thus result in what Filipa Melo Lopes calls “meaning vertigo” with respect to *Pr*’s social being, which is a “perception of a vertiginous and unsettling emptiness at the level of social meaning” that, “while [it] may not exactly track reality, it nevertheless constitutes a very real anxiety” that may prompt backlash on the part of *Pr* (Melo Lopes 2019, p. 2531).

### Amplificatory Challenges

If *Pr* do, sporadically, find themselves a place in counterhegemonic movements, this presents another challenge for a receptive public’s other, *amplificatory* function. If *Pr* are not to eventually capitulate to the broader *Pp* group dynamics that maintains an influence on their epistemic and material situation (e.g. through *akrasia* or ‘practical as well as sentimental reasons,’ as Eliot put it), *Pr* seeking to amplify counterhegemonic ideas or ‘signal-boost’ them to *Pp\Pr* (i.e. those not already similarly motivated to participate in receptive publics) run into a second, parallel dilemma:

*either* (Aa) *Pr* likens the colonial situation to others that *Pp* are collectively familiar with (e.g. sexism) and therefore apply to it the same analytical methods, judging it and the colonised in accordance with the traditional values *Pr* would have shared with *Pp*\*Pr* as *Pp*;

*or* (Ab) *Pr* and *Pp*\*Pr* find *Pp*’s traditional values and *Pr*’s new counterhegemonic values incommensurable, such that, first, *Pr* is unable to motivate *Pp*\*Pr* to solidarity and, second, *Pp*\*Pr* and *Pr* are no longer able to recognise each other as part of the same group—i.e. *Pp* splinters into *Pr* and *Pp*\*Pr*.

With (Aa), the same problem that we saw in horn (Da) is reiterated now at the interface between *Pr* and *Pp*\*Pr* instead of *Pr* and *Mr*, with the further caveat that the dynamics of a receptive public alone, now not just confronting *Pr* alone, are even less able to act as counterweight against appropriation by *Pp*\*Pr* [i.e. a Type 1 Failure]. This may not be a problem in a broader sense for the maintenance of counterhegemonic ideas by *Mc*, since—if we assume that *Pr does* give themselves over to counterhegemonic movements—*Pr* ∪ *Mc* is still a better outcome than *Mc* alone as counterweight. However, leading into the second horn, this would mean an inability for *Pr* to motivate *Pp*\*Pr* to political solidarity.

Consider, for example, how the aforementioned novel *Brights Celestials* by Straits Philosophical Society members Lamont and Tan was received in the imperial metropole of London (published under the pseudonym ‘John Coming Chinaman’ by Lamont). The novel followed the life of Tek Chiu, a ‘coolie’-turned-public-official from his hometown in Southern China, through Dutch Java, to Singapore, aiming to illuminate Chinese life both within China and abroad in the colonies, specifically in the context of Christian missionary efforts—unique at the time for offering such considerations through a Chinese narrative perspective. Unlike a conventional novel, its chapters were peppered with sociological observations focusing on topics under the British such as dehumanising practice of the ‘coolie’ trade, gender inequality in Chinese culture, Chinese secret societies, the uses and abuses of opium, and the transnational flesh trade. Lamont and Tan’s attempt to amplify a Chinese perspective on these concerns within the broader colonial public sphere, however, was received more as a cultural curiosity or fascinating postcard: it had a mixed reception among metropolitan reviewers, praised largely for its unique selling point, but panned for its style and relative optimism about the prospects of Christian missions among the Chinese.^8^ Further, between 1904 to 1910, the British Conservative government imported more than 60,000 ‘coolie’ labourers to work the gold mines in Witwatersrand to recuperate losses from the Second Boer War (see Yap and Man 1996). The subsequent backlash over this policy, which contributed strongly to the Liberal Party’s landslide victory over the Conservatives, was split between outrage over ‘Yellow Peril’ and ‘Chinese slavery’—the latter being more likely due to humanitarian reports of how Chinese labourers were treated rather than the efforts of the two Straits Philosophical Society members.

With (Ab), the antagonism between coloniser and colonised would be—even if initially intended for amelioration in *Pr\Mr* by Habgood-Coote et al.—exacerbated as the existential threat to *Pp* (first glimpsed by *Pr* as a developmental challenge) is materialised in the split between *Pp*\*Pr* and *Pr*. One way this concern can be illustrated is (famously) in *The Manifesto of the Community Party*, which imagines that “when the class struggle nears the decisive hour, the process of dissolution going on within the ruling class […] assumes such a violent, glaring character, that a small section of the ruling class cuts itself adrift, and joins the revolutionary class” (MECW 6: 494). That is, “the more or less veiled civil war, raging within existing society, [develops] up to the point where that war breaks out into open revolution” that aims at the “violent overthrow” of oppressors (MECW 6: 495).

While not involving the ‘violent overthrow’ of oppressors, we find a similar fragmentation of the extant public sphere to have occurred with certain members of the Straits Philosophical Society. Lamont returned to Scotland in 1896 (or 1897). It is unclear what exactly motivated Lamont to leave Singapore, between the withdrawal of support for his educational efforts in Singapore by the English Presbyterian Mission and his undertaking a BD (see Mouton 1989; Johnson 2001). But Lamont’s more liberal stand against racial prejudice was also incongruent with the colony being increasingly enamoured with racial hierarchy and the authoritarian approach in Dutch Java, *contra* the colony’s broader liberal commitments—as expressed by some colonial administrators who were also society members, e.g. the aforementioned Ridley (see Lim & Brophy 2023). Reportedly, for Lamont,

Race prejudice or colour question was not in the dictionary of his life. He mixed with all sections freely and his labour ideals marked the feeling of generosity, of sympathy and of uplift towards the working man of any class or creed. (“The late Mr Lamont” 1933, 193; quoted in Mouton 1989, 66)

Further, Lamont’s close collaborator Tan grew increasingly reclusive in his later life and withdrew from both the society and public-intellectual work (his last recorded contribution to the society being in 1915 and his last recorded public lecture being in 1916). Prior to this, in 1907, his testimony to the colonial Commission appointed to enquire into matters relating to the use of opium in the Straits Settlement and the Federated Malay States (1907–1908) takes on a clearly antagonistic tone. Tan rebukes the president’s proposed approach to opium as “an advocation of white men punishing the vices of coloured people, while tolerant of their own people,” before mockingly remarking:

Besides, fancy what Singapore would have done if it had not been for opium, how handicapped Sir Stamford Raffles [the founder of the colony] would have been if he had not this big revenue to hand [...] (Proceedings of Commission 1909, 296).

And, despite having been made the society’s president for a number of years, in 1921, Lim left the society and the colony, to take up an administrative position for a nascent Amoy [now Xiamen] University in Republican China—a move that rendered him “a radical” engaged in a “political and anti-British venture” in the eyes of the colony’s governor (Goh 2010, p. 503). This, importantly, occurred alongside the dissolution of the Straits Philosophical Society. Newspapers reported that Lim’s move was due to his frustrations with a ‘colour-bar,’ despite having been a volunteer in the imperial efforts of the First World War, as well as a (token) Chinese member of the colony’s legislature. In fact, earlier in 1919, Lim resigned and walked out from the legislature (whereof several other members of the Straits Philosophical Society were a part) in a desperate protest at an attempt to discourage the Chinese language being taught in the colony:

Oh, do not make me despondent, Sir! Do you want me to turn my eyes towards China? I feel this country [Singapore] is my own, and I feel there should be no line drawn. I can afford to pay for the education of my children, but there are many poor people who cannot pay for their children. […] Do you think that because you give us free education for four years in the Chinese language we are going to become Chinese patriots? […] Oh, do not be warped by this fear, this bogey of nationalism! […] How many of us can think of China? We can think more of our bowl of rice. (Legislative Council proceedings, 25 Oct. 1933, p. 190, LCPP 1933; quoted in Goh 2010, 504)

Whether the epistemic situation we are specifically concerned about is materialised in the same manner as imagined by Marx and Engels or in a whimper like the Straits Philosophical Society, the receptive public can nevertheless be thus seen to be able to occasion a breakdown in the extant public sphere.

## Troubleshooting Receptive Publics

Habgood-Coote, Ashton, and El Kassar’ goal with the introduction of ‘receptive publics’ was to shift the focus of academic discussions of political fragmentation away from a preoccupation with speech, and towards the topic of listening and understanding. They drew on literature on the public sphere and on contemporary social epistemology to argue for the importance of supporting two functions of a receptive public: the developmental and amplificatory functions.

In this paper, we have brought their work into contact with the historical archive and with philosophical literature on colonialism, in order to explore two challenges to these functions. We saw that in the face of such challenges, the goals of Marxian thinkers like Frantz Fanon are often more radical in tone than those set out by Habgood-Coote, Ashton, and El Kassar. Counterpublics are there meant to guide *counter-violence* against the violence of colonial publics, not merely *counter-speech*, or even failures to comprehend, and they focus on, straightforwardly, materially dissolving colonial publics and *a fortiori* the extant network of publics (cf. Srinivasan 2016), rather than on reconciling the world views that different publics have produced.

This suggests that receptive publics would be at best a *means* to catalyse such a dissolution. In *The Wretched of the Earth*, Fanon describes how the *colonised* intellectual may be disillusioned by their engagement within a receptive public before they find themselves reinvigorated by participating in acts of resistance that counter the instrumental and institutional violence of colonialism. It is in resistance, in a *material struggle*, that the colonised intellectual learns that undoing coloniality requires an abandonment of the colonial public and an assertion of a counterpublic not beholden to the former. The epistemic products of the counterpublic and material resistance, in dialectically enriching each other, would free such a counterpublic from its reactive nature to develop into a positive national culture, which includes revising fundamental ideals like humanity not within a receptive public but with the counterpublic’s resistance against hegemony.

Our paper reveals two lessons for receptive publics. First, if there is a role for receptive publics in the context of colonial struggle, it is not as a mere communicative intermediary for oppressed groups, but as a possible dialectical step that a counterpublic may take towards new modes of socio-material life. Historian Chua Ai Lin observes that despite the fact that “fundamental constitutional change [was] not achieved” with the conciliatory efforts of pre-Second World War intellectuals such as Lim (through failed receptive publics), the “self-confidence and ability to think politically” of “English-educated post-war politicians” who eventually led the decolonisation of Singapore, developed in the “atmosphere of vocal public opinion” of the Straits Chinese counterpublic in the “Asian Anglophone public sphere” (2008, p. 31). More immediately and in a more transnational context, Fermanis notes that, despite the *Straits Chinese Magazine*’s cessation in 1907, the “reformist agenda and belief in the importance of culture for Chinese identity” espoused by contributors like Straits Philosophical Society members Tan and Lim “was later mobilised by Sun Yat-sen in the service of a more aggressive kind of cultural nationalism and sinification—one that rested on a revitalised Confucianism as a distinctive form of religious modernity, and saw Chinese-language education both as a source of national strength and an effective response to the ongoing threat of western imperialism” (2021, p. 372). However—and this is the second lesson— if such new modes of socio-material life are to be developed alongside, rather than with the expulsion of, *Pr*, assisting the development of “novel and non-oppressive conceptualisations of [*Pr*’s] privileged identities” cannot be a mere possibility within receptive publics (as it is for Habgood-Coote et al. 2024), but is rather *a necessity*.

Whether or not such dialectical transformation then means that deliberative democracy and its very values of *liberté*, *égalité*, and *fraternité*—underpinning the three aspects of the Listening Problem that receptive public are meant to resolve—are up for revaluation lies outside the scope of this paper. As is the question of how much our conclusions carry over to oppressive contexts other than colonial ones. But what, we hope to have made clear is that equal, if not greater, attention must be paid to the material conditions of certain public networks (see, e.g., Harikrishnan 2020), as well as the transformations that systematically accompany the reception of certain counterhegemonic ideas therein.

## Footnotes

1 At least in its heyday, between roughly 2009–2022 (and before it was bought, restructured, and renamed to ‘X,’ by Elon Musk).

2 For discussions on the formation and dynamics of imperial public spheres, see Fraser 2014, Ataíde Fonseca 2024.

3 During this, an audacious (British) East Indian Company merchant and mid-tier official, James Flint, broke Qing court protocol and attempted to express his grievances over a business transaction directly to the Emperor.

4 James Legge, who is credited with translating the Chinese classics as part of Max Müller’s landmark *Sacred Books of the East* series (1879–1910), relied on the help of Chinese boys from British-controlled Sinophone territories—such as the Peranakan Chinese Song Hoot Kiam.

5 Charles Warren (1840–1927) was deeply entwined with the transnational affairs of the British Empire. He had been part of a group of British archaeologists in Palestine, as well as the head of the London Metropolitan Police during the Jack the Ripper murders, before he became a garrison commander in Singapore until he left to participate in the Second Boer War.

6 See also Wilson (2025) for a consideration of Tan’s Daoist idealism.

7 Tim Connolly also notes a third kind of incommensurability, *evaluative incommensurability*, where “there are no rational grounds for deciding whether a view from one tradition is superior to a view from another” (2023, p. 72), which can also be found in the dynamics of the Straits Philosophical Society.

8 The *Saturday Review of Politics, Literature, Science and Art* referred to “the diction of the volume [as] exactly that which we should expect [a Chinese “interpreter in the English Court at Singapore”] to employ—rigidly correct, but with a correctness which only emphasizes more forcibly the impression of an unEnglish origin” and “a love story [being] not the medium which we should, ourselves, have selected for conveying to an ignorant world some light on Chinese religion and government” (May 26, 1894, 559–60). *Colonies and India* referred to *Bright Celestials* as “a novel novel with a vengeance” for which if its “purpose had been kept a little more in the background it would have been better—much better”— especially in relation to its assessment “about the prospects of Chinese missionary enterprise” which “do not seem justified by facts” (March 10, 1894, 28).
